# A Palette of Minimally Tagged Sucrose Analogues for Real‐Time Raman Imaging of Intracellular Plant Metabolism

**DOI:** 10.1002/anie.202016802

**Published:** 2021-02-26

**Authors:** Fabio de Moliner, Kirsten Knox, Doireann Gordon, Martin Lee, William J. Tipping, Ailsa Geddis, Anke Reinders, John M. Ward, Karl Oparka, Marc Vendrell

**Affiliations:** ^1^ Centre for Inflammation Research The University of Edinburgh UK; ^2^ Institute of Molecular Plant Sciences The University of Edinburgh UK; ^3^ Cancer Research (UK) Edinburgh Centre The University of Edinburgh UK; ^4^ EaStCHEM School of Chemistry The University of Edinburgh UK; ^5^ Centre for Molecular Nanometrology University of Strathclyde UK; ^6^ Department of Plant and Microbial Biology University of Minnesota USA

**Keywords:** imaging, probes, stimulated Raman scattering, sugars, transport

## Abstract

Sucrose is the main saccharide used for long‐distance transport in plants and plays an essential role in energy metabolism; however, there are no analogues for real‐time imaging in live cells. We have optimised a synthetic approach to prepare sucrose analogues including very small (≈50 Da or less) Raman tags in the fructose moiety. Spectroscopic analysis identified the alkyne‐tagged compound **6** as a sucrose analogue recognised by endogenous transporters in live cells and with higher Raman intensity than other sucrose derivatives. Herein, we demonstrate the application of compound **6** as the first optical probe to visualise real‐time uptake and intracellular localisation of sucrose in live plant cells using Raman microscopy.

## Graphical abstract

We describe a chemical strategy to prepare sucrose derivatives with vibrational tags and the characterisation of the first optical analogue of sucrose able to image real‐time trafficking of sucrose in live plant cells.

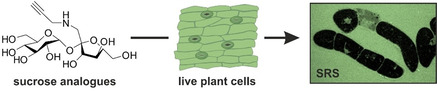

Sucrose metabolism is one of the main processes that regulates the development, growth and functioning of higher plants.^[1]^ However, imaging studies of sucrose metabolism are scarce compared to other saccharides, such as glucose, fructose, sialic acid or lignin components.^[2]^ Sucrose is used for energy storage in plant cells^[3]^ and for the long‐distance transport of carbon fixed during photosynthesis.^[4]^ As a result, sucrose is the most abundant sugar in plants, with concentrations in the phloem around 100 mM,^[5]^ and is pivotal in energy metabolism, signalling and gene expression.^[6]^ Although the mobilisation and intracellular trafficking of sucrose are essential processes in plant metabolism, there are no optical probes that can image its cell uptake and localisation with high spatiotemporal resolution.

Several different techniques have been employed to investigate the distribution of sucrose in plants.^[7]^ HPLC‐MS is commonly used but limited by the lack of spatial resolution and its destructive nature.^[8]^ Assays with sucrose bearing radioactive isotopes (e.g., ^14^C and ^3^H)^[9]^ suffer from limited spatial and temporal resolution as well as intrinsic drawbacks including the need for safety precautions. Coinciding with the emergence of optical probes as inexpensive tools for the non‐invasive monitoring of biological events,^[10]^ sucrose analogues including fluorescent labels have been recently reported.^[11]^ These molecules recapitulate some biological features of the native metabolites, but their large size hampers their application for monitoring intracellular localisation and metabolism in live cells.

Stimulated Raman scattering (SRS) enables the detection of chemical groups in their native environment.^[12]^ The increased sensitivity of SRS has been used to visualise metabolites and small‐molecule drugs in live cells and plants.^[13]^ Among others, SRS imaging has been reported to study glucose[^2c^, ^14^] and cholesterol^[15]^ metabolism, protein synthesis and degradation,^[16]^ DNA synthesis,^[17]^ lipid dynamics in cell membranes^[18]^ and the chemical composition of bacterial cell surfaces (Figure 1).^[19]^ Furthermore, chemical labels for SRS are smaller than fluorophores, causing minimal disruption in the molecular recognition properties of native metabolites. Given the capabilities of SRS imaging for monitoring the distribution of small metabolites in live cells, we have designed a chemical strategy to synthesize Raman‐active sucrose analogues for direct metabolic imaging in live plant cells. Herein we report a collection of derivatives including very small vibrational tags (Figure 1), obtained with good yields in three synthetic steps. Following their spectroscopic evaluation for biological SRS imaging, we have characterised the transport of an alkyne‐tagged sucrose in genetically modified yeast cells. Finally, we have used our alkyne‐tagged sucrose to image for the first time how sucrose translocates in live plant cells.

**Figure 1:**
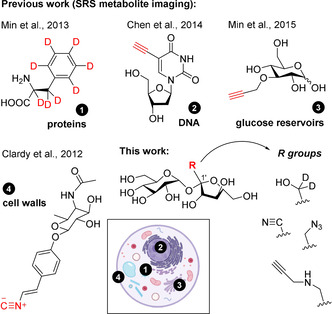
Previous and current work for labelling of small metabolites using vibrational tags for SRS imaging of intracellular metabolism.

Label‐free SRS imaging can resolve cellular structures by exploiting inherent vibrational groups of target molecules.^[20]^ Alternatively, chemical tags can be incorporated to improve detectability and sensitivity. Groups vibrating within the silent window of the Raman spectrum (i.e., from 2000 to 2500 cm^−1^) are optimal to minimise background interference from cellular components (e.g., proteins, nucleic acids, fatty acids).^[21]^ Different chemical groups (e.g., alkynes^[22]^ and nitriles^[23]^) have been reported for Raman imaging (Figure 1). However, none of these have been reported for the derivatisation of sucrose or for in situ metabolic imaging in live plants. In this work, we synthesized a family of sucrose analogues with different bioorthogonal vibrational tags that could retain the properties of sucrose and be used to image intracellular mobilisation under physiological conditions by SRS microscopy.

Selective transformations involving one single position of a saccharide are a major challenge in carbohydrate chemistry^[24]^ because of the presence of multiple hydroxyl groups with similar reactivity.^[25]^ Bearing this in mind, we designed a general synthetic approach to introduce different chemical labels (e.g., azides, alkynes, nitriles) into sucrose with the minimal number of synthetic steps (Scheme 1). For this purpose, the identification of a single and accessible common precursor was crucial. We selected the perbenzoylated compound **1** as a suitable starting material because it possesses a free hydroxyl group in the position 1 of the fructose moiety, which has been reported for successful sucrose functionalisation,[^26^, ^27^] and also a single protecting group (i.e., benzoyl) orthogonal to the different Raman tags. First, we activated the free hydroxyl group in compound **1** as a triflate to render common reactive intermediate **2**. Nucleophilic substitution with sodium azide and final deprotection in basic media afforded the azido‐functionalised sucrose **4** with good overall yields (>50 % over 3 steps). Next, we investigated the installation of an alkyne tag. Initially, we evaluated acetylide as the smallest alkyne group, but its introduction proved unfeasible. In fact, trimethylsilyl acetylene failed to react with the activated triflate **2**, both under Sonogashira coupling conditions and after treatment with a strong base. Attempts to generate the alkyne by homologation with the Ohira‐Bestmann reagent were also unsuccessful (Supplementary Figure 1). Therefore, we employed propargylamine as an alkyne‐containing nucleophile using an activation/substitution/deprotection sequence to obtain the sucrose analogue **6**. Finally, the synthesis of a nitrile‐functionalised sucrose analogue was designed. Nucleophilic substitutions between the triflate intermediate **2** and sources of cyanide anion (e.g., trimethylsilyl cyanide, potassium cyanide) were attempted but unsuccessful (Supplementary Figure 2). Alternatively, we oxidised compound **1** with Dess–Martin periodinane and the resulting aldehyde **7** was reacted with hydroxylamine to produce the oxime **8**. The dehydration step afforded the protected nitrile‐functionalised sucrose **9** but the final deprotection under different reaction conditions did not afford compound **10** due to the poor stability of the tertiary nitrile group (Supplementary Table 1). Therefore, we included a short spacer between sucrose and the nitrile tag by reaction between the reactive intermediate **2** and the commercial aminopropionitrile. Importantly, this strategy afforded the nitrile **12** in high purity upon deprotection of the benzoyl groups.

**Scheme 1:**
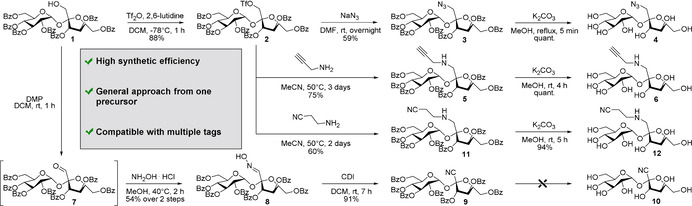
General synthetic strategy for the synthesis of Raman‐active sucrose analogues.

Before measuring the Raman properties of our sucrose analogues [i.e., compound **4** (azide), compound **6** (alkyne) and compound **12** (nitrile)], we acquired the Raman spectrum of pellets from the widely used tobacco BY‐2 plant cells.^[28]^ As shown in Figure 2, the spectrum of BY‐2 cells featured peaks found in many macromolecules (i.e., 1000 cm^−1^ for phenylalanine ring breathing, 1655 cm^−1^ for amide C=O stretching and 2845 cm^−1^ for C−H stretching from lipids and proteins) and a silent region between 2000 cm^−1^ and 2500 cm^−1^ (Figure 2 a). This observation confirmed the utility of Raman microscopy for detecting specific labels in plant cells with minimal background signal and maximum sensitivity. Next, we acquired the spectra of all sucrose analogues and compared them to the commercially available deuterated sucrose (**13**, Figure 2 b). We acquired the spontaneous Raman spectra of all derivatives in water and under the same experimental conditions to compare the wavenumbers and relative intensities of their Raman peaks. Notably, all four compounds displayed detectable peaks within the silent region (Figure 2 b), which were not present in native sucrose (Supplementary Figure 3). Deuterated sucrose showed a broad peak around 2115 cm^−1^, consistent with the literature data for carbon‐deuterium bonds.^[29]^ The azide derivative **4** showed a weak peak at 2113 cm^−1^, likely due to the reduced polarizability of the nitrogen‐nitrogen triple bonds.^[30]^ The Raman peak for the nitrile‐functionalised sucrose **12** was observed around 2250 cm^−1^. Finally, the alkyne‐tagged sucrose **6** displayed a strong Raman peak at 2116 cm^−1^, showing the highest intensity among all analogues. Compound **6** also showed a lower limit of detection in water (29 mM) than the commercial compound **13**, which is >100 mM (Figure 2 c and Supplementary Figure 4). Given these results and the chemical similarities between compound **6** and native sucrose [i.e., 342 Da vs. 379 Da (**6**), clog *P* −3.75 vs. clog *P* −3.52 (**6**), Figure 2 c], we proceeded to investigate its application as an optical probe for imaging sucrose uptake and intracellular localisation in live cells.

**Figure 2:**
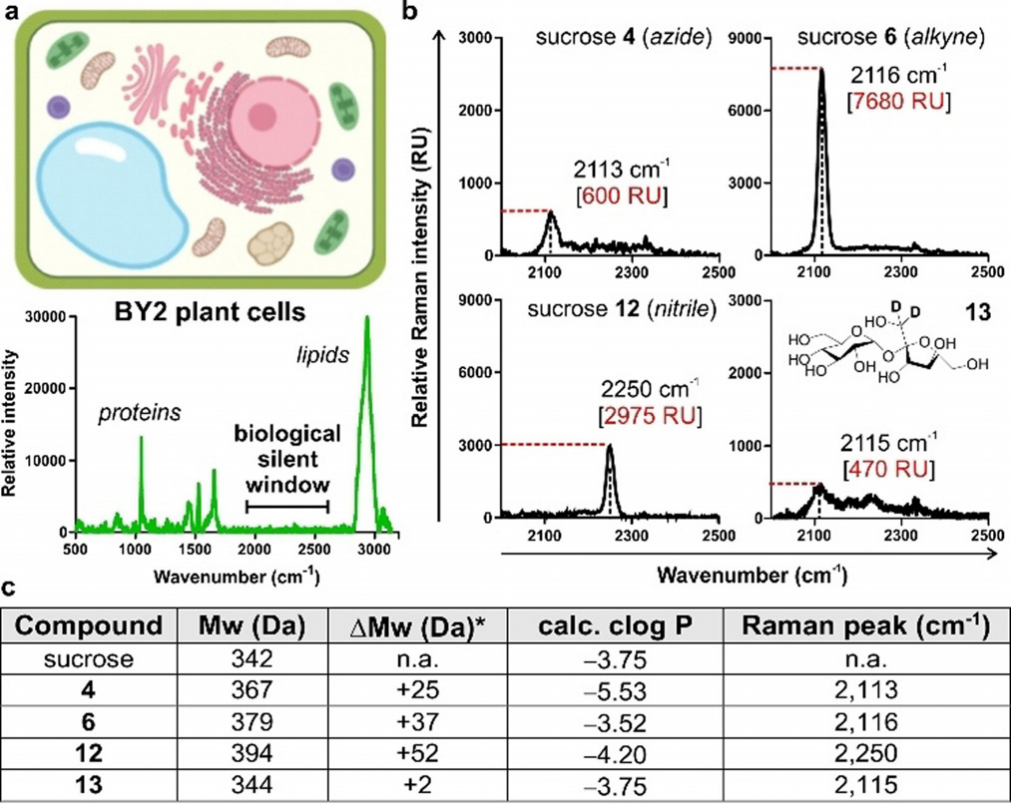
a) Schematic illustration of a plant cell and Raman spectrum of BY2 cells. b) Representative Raman spectra (3 independent experiments) of sucrose analogues (**4**, **6**, **12** and **13**) in water. Wavenumber and maximum intensity of the highest Raman peaks are highlighted in black and red, respectively. c) Summary of physicochemical properties of native sucrose and the synthesized sucrose analogues. *Differences in molecular weight between the synthesized analogues and native sucrose.

In order to study the uptake of the alkyne‐tagged sucrose analogue **6** in live cells, we utilised genetically modified yeast cells (SEY6210) that expressed sucrose transporters from different plant species (i.e., StSUT1, OsSUT1 and AtSUC2).^[31]^ For this purpose, we optimised an optical assay to measure the amount of compound **6** that had been internalised by each yeast line via derivatisation with the fluorogenic 3‐azido‐7‐hydroxycoumarin, which produces bright fluorescence emission around 460 nm after the cycloaddition reaction (Figure 3 a). This assay allowed us to rapidly compare the uptake of compound **6** through different transporters without the need for specialised equipment as in radioactivity experiments.[^9b^, ^32^] As shown in Figure 3 b, we observed much higher uptake of compound **6** in cells that expressed OsSUT1 and StSUT1 transporters over those expressing AtSUC2 transporters. Experiments in the same yeast cells transformed with the vector control pDR196 but lacking any SUT1 transporters instead showed very little uptake of compound **6** (Supplementary Figure 5). Whereas these results do not exclude the possibility that other transporters found in plant cells (e.g., SWEET transporters^[33]^) may recognise compound **6**, they show that alkyne‐modified sucrose can be taken up by type I (StSUT1) as well as type II (OsSUT1) transporters. To the best of our knowledge, compound **6** represents the first optical probe to monitor sucrose uptake in monocots (e.g., rice, maize, wheat), which express type II sucrose transporters. Furthermore, we analysed the degradation and functionality of probe **6** by measuring reactivity against sucrose invertase and cellular influx of Ca^2+^ induced by sucrose uptake. Our results show that probe **6** is only partially degraded by sucrose invertase (Supplementary Figure 6) and that its transport leads to increased levels of intracellular Ca^2+^, consistently with previous reports^[34]^ (Supplementary Figure 7).

**Figure 3:**
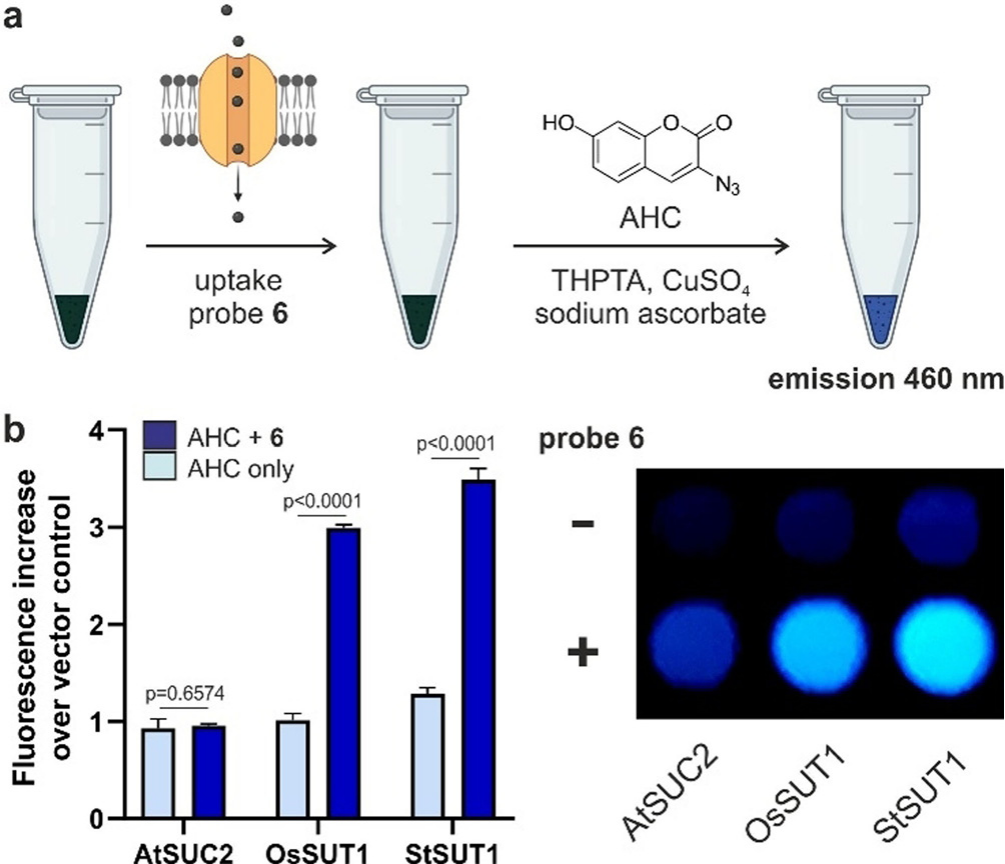
a) Schematic cell‐based assay for measuring sucrose uptake in yeast mutants. 5×10^5^ cells were incubated with compound **6** for 2 h at 30 °C, then centrifuged to obtain cell pellets that were incubated for 20 min with 3‐azido‐7‐hydroxycoumarin (AHC, 1 mM), THPTA (1 mM), CuSO_4_ (10 mM) and sodium ascorbate (10 mM). b) Fluorescence analysis (*λ*_exc_=400 nm, *λ*_em_=460 nm) of cell pellets containing SUC2 or SUT1 sucrose transporters after incubation or not with compound **6** (100 mM) and subsequent reaction with AHC. Fold increase relative to the vector control lacking sucrose transporters presented as means±SD (*n*=3). *P* values from one‐way ANOVA with multiple comparisons.

We finally examined the utility of compound **6** for real‐time imaging of sucrose trafficking in live plant cells. For these experiments, we incubated BY2 cells with compound **6** before performing SRS imaging in a multi‐photon microscope. Compound **6** showed no cytotoxicity (Supplementary Figure 8) and time‐lapse images were acquired over 30 minutes with simultaneous monitoring of the alkyne‐tagged compound **6** (2116 cm^−1^) and label‐free Raman signatures of plant cells (2930 and 2855 cm^−1^ for CH_3_ and CH_2_ groups, respectively) (Figure 4). We observed that compound **6** was gradually taken up by each cell at a time, with sucrose being initially localised in the cytoplasm. After 30 minutes, all BY2 cells had taken compound **6** up and equilibrated the intracellular sucrose concentration with that found in the media (i.e., 125 mM). Notably, most cells displayed uniform signal intensity throughout the cell, including the vacuoles. This observation indicates that compound **6** can enter the vacuoles, where plant cells store excess sucrose. Competition experiments with sucrose remarkably reduced the transport of compound **6** to the vacuoles, as shown in Supplementary Figure 9 and Supplementary Movies 1–2.

**Figure 4:**
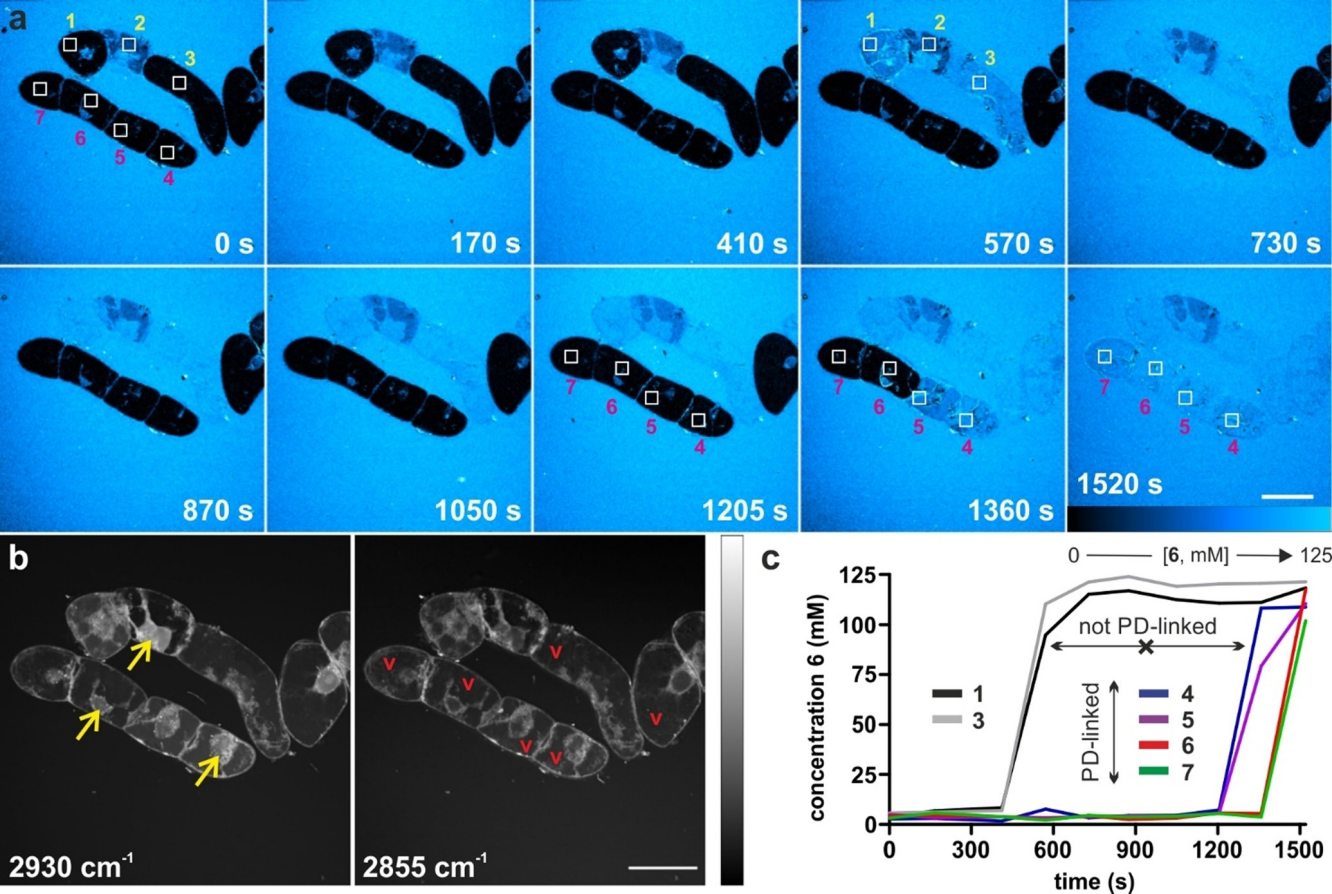
Representative live‐cell SRS images (from 3 independent experiments) of BY2 cells showing the uptake of the alkyne‐tagged compound **6** in real time. a) Time‐lapse (time in seconds) pseudo‐color Raman images (2116 cm^−1^) of BY2 cells upon incubation with compound **6**. Squares defining 7 different regions of interest to monitor the uptake and accumulation of sucrose in cells. Scale bar=50 μm. b) Label‐free SRS images at 2930 cm^−1^ (CH_3_ groups) and 2855 cm^−1^ (CH_2_ groups) highlighting the structures of BY2 cells, with vacuoles (red v) that confine the cytoplasm and organelles in the vicinity of the nucleus (yellow arrows). Scale bar=50 μm. c) Time‐course Raman intensity in different cells [ROIs in (a)] showing likely intercellular translocation across plasmodesmata (PD). Scale bar=50 μm.

Furthermore, BY2 cells are connected to each other through selective channels called plasmodesmata (PD). Our SRS images indicate that compound **6** could rapidly translocate across PD‐connected cells, like native sucrose does. This is suggested in Figure 4 a, where compound **6** appears to diffuse from cell 2 to cells 1 and 3, and where the PD‐linked cells 4–7 sequentially display bright intracellular Raman signals between 1205 and 1520 seconds. Altogether, these results validate the alkyne‐tagged compound **6** as the first optical probe to visualise in situ plant sucrose transporters that are prevalent in many crop species in real time.

In summary, we report the first Raman‐active sucrose analogue and its application for real‐time imaging of metabolic uptake in live plant cells. We have optimised an efficient synthetic approach for the generic preparation of sucrose derivatives including a variety of minimal vibrational tags (e.g., alkyne, azide, nitrile) providing readouts within the silent window of the Raman spectrum. Among these, the alkyne‐containing compound **6** showed strong Raman peaks, with remarkably high intensity and capacity to be recognised by type I and type II plant sucrose transporters. We demonstrated the utility of compound **6** to image in situ the intracellular localisation of sucrose in live plant cells, opening multiple opportunities for imaging metabolite trafficking and intracellular storage with enhanced spatial and temporal resolution.

## Conflict of interest

The authors declare no conflict of interest.

## Supporting information

As a service to our authors and readers, this journal provides supporting information supplied by the authors. Such materials are peer reviewed and may be re‐organized for online delivery, but are not copy‐edited or typeset. Technical support issues arising from supporting information (other than missing files) should be addressed to the authors.

Supplementary

Supplementary

Supplementary
